# Pathophysiology of SARS-CoV-2 infection in patients with intracerebral hemorrhage

**DOI:** 10.18632/aging.103511

**Published:** 2020-07-07

**Authors:** Sisi Dong, Peipei Liu, Yuhan Luo, Ying Cui, Lilong Song, Yingzhu Chen

**Affiliations:** 1 The Second Xiangya Hospital, Central South University, Changsha 410011, Hunan, China; 2 Department of Neurology, Clinical Medical College, Yangzhou University, Yangzhou 225001, Jiangsu, China; 3 Department of Neurology, Clinical Medical College of Yangzhou, Dalian Medical University, Yangzhou 225001, Jiangsu, China

**Keywords:** SARS-CoV-2, COVID-19, intracerebral hemorrhage, ACE2, Ang (1–7)

## Abstract

Intracerebral hemorrhage (ICH) is associated with old age and underlying conditions such as hypertension and diabetes. ICH patients are vulnerable to SARS-CoV-2 infection and develop serious complications as a result of infection. The pathophysiology of ICH patients with SARS-CoV-2 infection includes viral invasion, dysfunction of the ACE2–Ang (1–7)–MasR and ACE–Ang II–AT_1_R axes, overactive immune response, cytokine storm, and excessive oxidative stress. These patients have high morbidity and mortality due to hyaline membrane formation, respiratory failure, neurologic deficits, and multiple organ failure.

## INTRODUCTION

Novel coronavirus (2019-nCoV)–associated pneumonia cases first appeared in Wuhan, Hubei Province, China, in December 2019 [1]. Whole-genome sequencing identified a novel coronavirus—severe acute respiratory syndrome coronavirus 2 (SARS-CoV-2) [2, 3]. In the following months, SARS-CoV-2 rapidly spread throughout China and the world. By May 26, 2020, SARS-CoV-2 had resulted in 84,543 infections and 4,645 deaths in China, as reported by National Health Commission of the People’s Republic of China. In addition, other countries reported 5,468,627 confirmed cases and 345,544 deaths. The World Health Organization declared SARS-CoV-2 a public health emergency and named the virus Corona Virus Disease 2019 (COVID-19).

Although the source of SARS-CoV-2 and its pathogenesis are still being studied, COVID-19 is a systemic disease that can lead to pneumonia, respiratory failure, and acute respiratory distress syndrome (ARDS) and has high morbidity and mortality. COVID-19 also affects the cardiovascular, renal, cerebrovascular, and blood coagulation systems. Genome sequencing of patients’ cerebrospinal fluid has identified the presence of SARS-CoV-2 in the brain, which is also seen in SARS and Middle East respiratory syndrome (MERS) infection [4]. Here, we review the pathophysiology of SARS-CoV-2 infection in patients with intracerebral hemorrhage (ICH).

## Characteristics of SARS-CoV-2

Coronaviruses (CoVs), part of the subfamily *Orthocoronavirinae* in the family *Coronaviridae* of the order Nidovirales, are enveloped, nonsegmented, positive-sense, single-stranded RNA viruses [5]. Some CoVs are transmitted from animals to people and have gradually developed as pathogens of the respiratory, gastrointestinal, and central nervous systems in human. Examples include SARS, which caused an outbreak in 2002, and MERS, which caused an outbreak in 2012, both of which affect the lower respiratory tract [6, 7]. Genome sequencing has identified 2019-nCoV as a *Betacoronavirus*. SARS-CoV-2 and two bat-derived SARS-like strains, ZC45 and ZXC21, form an independent clade within lineage B of the subgenus *Sarbecovirus* [8, 9]. The two bat SARS-related coronaviruses closest to SARS-CoV-2, ZXC21 and ZC45, can infect suckling rats and cause brain tissue inflammation and pathological changes in the lung and intestine [10].

SARS-CoV-2 contains a single positive-sense RNA genome and is around 60 to 140 nm in diameter [5]. The genome sequence of SARS-CoV-2 has 89% nucleotide identity with the bat SARS-like CoV ZXC21, 86.9% with the bat SARS-like CoV ZC45, and 82% with the human SARS-CoV [10–12]. The phylogenetic trees of SARS-CoV-2’s orf1a/b, spike, envelope, membrane, and nucleoprotein also cluster closely with those of the bat, civet, and human SARS coronaviruses [10, 11, 13]. However, the external subdomain of spike’s receptor binding domain in SARS-CoV-2 shares only 40% amino acid identity with other SARS-related coronaviruses [8, 10].

SARS-CoV-2, like SARS-CoV, manipulates angiotensin-converting enzyme 2 (ACE2) as the viral receptor and invades type 2 alveolar epithelial cells in the lower respiratory tract [11]. ACE2 inhibitors prevent SARS coronavirus from constant viral replication in Vero E6 cells [14]. The receptor binding domain on the S1 subunit of the SARS-CoV-2 spike protein (S glycoprotein) and the transmembrane domain of ACE2 are implicated in SARS-CoV-2 infection [2, 15].

A majority of the earliest confirmed patients infected with SARS-CoV-2 were exposed to wild animals sold in the Huanan Seafood Wholesale Market. Although it is difficult to pinpoint the exact source or the intermediate host of the novel coronavirus, the first cluster of pneumonia cases suggests that person-to-person transmission via the respiratory route occurred [16]. The digestive system is also hypothesized to be a route of SARS-CoV-2 transmission.

## ICH patients are vulnerable to SARS-CoV-2 infection and develop serious complications as a result of infection

The general population is susceptible to SARS-CoV-2 infection. As of February 11, 2020, the Chinese Center for Disease Control and Prevention had identified 72,314 cases of COVID-19, including 55,239 confirmed patients, 16,186 suspected infections, and 889 infections without any symptoms [17]. 87% of the patients are between 30 and 79 years old. Clinical symptoms at the beginning of COVID-19 infection include chills, fever, cough, fatigue, myalgia, dyspnea, and diarrhea. Chest computed tomography (CT) images show ground-glass opacity in both lungs and, in severe cases, progressive consolidation of multiple lobular and subsegmental tracts. However, many infected patients are asymptomatic and have normal chest CT scans. Asymptomatic patients with SARS-CoV-2 infection, as well as those with atypical neurologic manifestations such as headache, dizziness, nausea, and vomiting, contribute to misdiagnosis and delayed treatment. According to the Chinese Center for Disease Control and Prevention, 81% of the 72,341 patients diagnosed with COVID-19 had mild disease, and the mortality rate was approximately 2.3%. However, the fatality rate increased to 8.0% in people age 70 to 79 years old and 14.8% in those age 80 or older. Infected patients with underlying diseases also had higher fatality rates: 10.5% in patients with cardiovascular disease, 7.3% for diabetes, 6.0% for hypertension, and 5.6% for cancer. A review of the clinical features of 138 confirmed patients in Zhongnan Hospital of Wuhan University confirmed that ICU patients were obviously elder and were more likely to have underlying diseases, as well as having higher risk for poor outcome [18]. Therefore, the worst complications and outcomes occur in older patients and those with chronic diseases, such as pulmonary disease, diabetes, hypertension, heart failure, atherosclerosis, cerebrovascular disease, and cancer. Patients with severe SARS-CoV-2 infection develop pneumonia and extrapulmonary pathological changes. Complications in patients with severe infection include hypoxemia, pulmonary edema, ARDS, postviral bacterial superinfection, septic shock, metabolic acidosis, blood coagulation dysfunction, and multiple organ damage. A retrospective, single-center study of 99 cases of COVID-19 in Wuhan Jinyintan Hospital revealed that severe patients had high levels of alanine aminotransferase (ALT), aspartate aminotransferase (AST), myocardial zymogram, blood urea nitrogen and serum creatinine, all of which were implicated with multiple organ damage. Biopsy samples of tissues from patients with SARS-CoV-2 indicate impairment of alveolar epithelial cells and pneumocytes in both lungs, exudation of extracellular fluid in alveolus, infiltration of lymphocytes and macrophages, and formation of hyaline membrane, indicating ARDS, which also occurs in SARS and MERS coronavirus infection [19, 20].

ICH accounts for 20% to 30% of strokes in China and is associated with high mortality and morbidity, with most survivors experiencing neurologic and cognitive impairment. The physiological status go to the bad with age and elder persons have higher possibility to develop underlying diseases, consisting of hypertension, diabetes, and dysfunction of blood coagulation, all of which are interact with the occurrence and development of ICH [21]. Hypertension is the mainly risk factor of ICH, as well as amyloid angiopathy, hemangioma, arteriovenous malformations, coagulopathy, and cerebroma [22]. Therefore, ICH is associated with old age and underlying conditions such as hypertension and diabetes. ICH patients, susceptible to SARS-CoV-2 infection, are prone to develop serious complications and need ICU admission.

ICH exerts mass effect and causes primary physical damage that is dependent on the location, volume, and expansion of the hematoma. Secondary injury is caused by brain edema, the inflammatory cascade, and hematoma decomposition products. After the interaction between SARS-CoV-2 and the ACE2 receptor, some infected patients rapidly develop elevated blood pressure, which brings about severe cerebral changes, including activated microglia, accumulated ferritin, damaged neurons, and impaired neurologic function [23]. One report describing 41 cases of COVID-19 indicated that prolonged prothrombin time, elevated D-dimer, and severe platelet reduction occur in ICU patients with SARS-CoV-2 infection [24]. Then ICH patients may develop blood coagulation dysfunction as a result of infection. The high levels of thrombin is a trigger of early perihematomal brain edema; thrombin affects a variety of cells, including microglia, neurons, and brain endothelial cells, and destroys the blood–brain barrier (BBB) [25]. Low platelet activity is a marker of severe ICH, and platelet transfusion in the acute phase can limit hemorrhage volume and attenuate poor outcomes [26]. The BBB inhibits cerebrum invasion, regulates substantial exchange, and maintains homeostasis in the center nervous system. The viral invasion and breakdown of the BBB results in immunocyte recruitment in the central nervous system. Overactivation of the immune response and proinflammatory factors can lead to cellular apoptosis and necrosis, endothelial impairment, brain edema, and neuronal loss. In detail, the pathophysiology of ICH patients with SARS-CoV-2 infection includes viral invasion, dysfunction of the ACE2–Ang (1–7)–MasR and ACE–Ang II–AT_1_R axes, overactive immune response, cytokine storm, and excessive oxidative stress.

## SARS-CoV-2 brain invasion and ACE2

The renin-angiotensin system (RAS) consists of the protease renin, angiotensinogen, angiotensin-converting enzyme (ACE), and angiotensin II. The local brain RAS includes angiotensinogen, peptidases, angiotensins, and specific receptor proteins that play specific roles in development of cerebrovascular disease [27, 28]. ACE2, a homologous enzyme of ACE, is secreted by endothelia and smooth muscle cells. A study pointed that SARS-CoV-2 can manipulate all but mouse ACE2 as the entry receptor in the ACE2-expressing cells, which might permit the viral invasion and replication in multiple organs. ACE2 is found in arterial and venous endothelial cells and arterial smooth muscle cells in most organs, including oral and nasal mucosa, nasopharynx, lung, stomach, small intestine, colon, skin, lymph nodes, thymus, bone marrow, spleen, liver, kidney, and brain [14, 29].

Pathologists obtained human brain tissue from autopsies and research on the staining for ACE2; endothelial and smooth muscle cells of cerebrum were stained [14]. The barrier between plasma and brain cells is formed by brain capillary walls and glial cells and the barrier between plasma and cerebrospinal fluid is formed by choroid plexus. The expression of ACE2 in endothelial and smooth muscle cells allow viral invasion and replication in the blood-brain barrier. The BBB breakdown includes swelling of endothelial cells, necrosis, apoptosis, inflammatory injury and systemic vasculitis. Genome sequencing of patients’ cerebrospinal fluid confirmed SARS-CoV-2 infection in the brain [17]. The infected patients with atypical neurologic manifestations such as headache, dizziness, nausea, and vomiting are important signs for SARS-CoV-2 brain invasion. In addition, autopsies from patients with SARS infection have detected SARS-CoV particles and genomic sequence in cerebral neurons, as well as in T lymphocytes and monocytes in the circulating blood of multiple organs [30]. After intranasal inoculation of MERS-CoV in transgenic mice, study of brain tissues indicated viral invasion. Mice infected with the JHM and A59 strains of murine hepatitis virus (MHV) manifest an acute encephalomyelitis and gradually develop demyelinating disease as a result of persistent viral stimulation. In addition to pulmonary disease, coronaviruses also cause pathological changes in the cerebrum due to their neuroinvasive and neurotropic properties [31].

## SARS-CoV-2 and the dysfunction of the ACE2–Ang (1–7)–MasR and ACE–Ang II–AT_1_R axes

Angiotensin 1-7, which is transferred by endopeptidases, ACE2, and ACE from angiotensin I, binds to the Mas receptor and is an effective and protective vasodilator [32]. Mas receptors are distributed throughout the brain, including the medulla and forebrain, which are associated with cardiovascular regulation, and the hippocampus, amygdala, anterodorsal thalamic nucleus, cortex, and hypoglossal nucleus [33]. In contrast to the effects of Ang II in the brain, Ang-(1-7) regulates the cardiovascular reflex and mediates blood pressure by releasing nitric oxide (NO) and activating the PI3K-Akt-PKB pathway [34]. The interaction between Ang-(1-7) and the Mas receptor decreases reactive oxygen species (ROS) production by cleaving Ang II or inhibiting AT_1_ receptors [35]. Ang-(1-7) and the G-protein-coupled receptor Mas, which initiate the release of cytokines and activate and recruit leukomonocytes, reduce inflammation by the restraining Des-Arg9 bradykinin (DABK)-mediated pathway [36, 37]. The ACE2–Ang-(1-7)–Mas axis is a protective regulator in the center nervous system; it regulates blood pressure and inhibits inflammatory injury, oxidative stress, fibrosis, and cellular apoptosis [38, 39]. Injection of Ang-(1-7) in the ventricle of rats reduces ICH-induced injury, resulting in limited hematoma expansion, decreased microglia, and neuronal recovery [40]. In addition, administration of Ang-(1-7) in mice with aneurysmal rupture inhibits the production of TNF-α and IL-1β and attenuates pathological damage [41]. The ACE2–Ang (1–7)–MasR axis and ACE–Ang II–AT_1_R axis counterbalance each other to maintain cerebral homeostasis [42]. Thus, pathological disruption of ACE2 and Ang II can result in neurologic damage [43].

Infection and endocytosis of SARS-CoV-2 particles downregulate active ACE2 and Ang-(1-7) and increase Ang II. The subsequent inhibition of the ACE2–Ang (1–7)–MasR axis and overactivation of the ACE–Ang II–AT_1_R axis underlie the progressive pathological deterioration in the cerebrum seen in patients with SARS-CoV-2. Disruption of the ACE–Ang II–AT_1_R axis contributes to rapidly elevated blood pressure [44]. Stimulation and production of Ang II in local brain, which binds to AT_1_ receptors, activates the inflammatory NF-κB pathway and superoxide production by activating nicotinamide adenine dinucleotide phosphate (NADPH) oxidase [45]. Increased ROS production damages brain tissue, which is full of polyunsaturated fatty acid. In addition, overactivation of ACE–Ang II–AT_1_R is partly responsible for brain inflammation and cellular apoptosis and necrosis, leading to endothelial impairment, brain edema, and neuronal injury. Administration of brainc Ang II receptor inhibitor attenuated acute inflammatory responses in an animal model with bacterial infection [46]. The brain inflammation with positive feedback seen in ICH patients with SARS-CoV-2 infection, which is postulated to be a result of dysfunction of the ACE2–Ang (1–7)–MasR and ACE–Ang II–AT_1_R axes, results in excessive oxidative stress and elevated cytokines, chemokines, and toxic substances, which lead to neuronal injury, cell death, brain edema, and neurologic deficits. Hematoma expansion and brain edema contribute to physical pressure on neighboring structures, such as arterial vessels, the aqueduct of Sylvius, and the brainstem, leading to cerebral ischemia, obstructive hydrocephalus, cardiorespiratory dysfunction, intracranial hypertension, and even cerebral hernia.

## SARS-CoV-2, immune evasion and overactivated immune responses

Among hospitalized patients with SARS-CoV-2 infection, general laboratory abnormalities include leukopenia and lymphopenia. These abnormalities indicate that both the viral burden and the reaction of immune system play a critical role in SARS-CoV-2 invasion and replication. The immune system can inhibit coronavirus, clean up apoptotic cells, and promote tissue recovery in the cerebrum. Chemotactic factors help leukocytes migrate to the correct position to fight infection, and abnormal secretion can aggravate the cerebral immunopathology. Conversely, weak immune systems and insufficient immune responses are associated with viral survivors and rapid coronavirus invasion. Therefore, the relationship between SARS-CoV-2 infection and the immune response needs to be investigated, with potential measures provided to interfere with viral dissemination, clear the virus, and reduce tissue impairment.

After the internalization of coronavirus particles, host cells recognize the coronavirus and initiate an innate and adaptive immune response against the viral infection; the complement system is also activated. Interaction between cell-surface pattern recognition receptors (PRRs) and pathogen-associated molecular patterns (PAMPs), activation of proinflammatory signaling proteins and pathways, production and release of several inflammatory factors, and migration of immunocytes occur in the immune and inflammatory settings [36]. In addition, complementary autocrine and paracrine signaling ensures that the infected cells and surrounding uninfected cells express a series of interferon-stimulated genes (ISGs), which establish an antiviral microenvironment [47]. The PRRs in host cells that detect pathogens contain toll-like receptor (TLR), RIG-I-like receptors (RLRs), NOD-like receptors (NLRs), C-type lectin-like receptor (CLR), cytoplasmic DNA receptor (CDR), type I interferons (IFNs), and dendritic cells (DCs) and restrict viral pervasion with the help of macrophages, natural killer cells, T/B cells, and immune molecules [48]. The main function of macrophages is to phagocytose and digest cell debris and pathogens and activate lymphocytes or other immune cells in response to pathogens. Macrophages and DCs infected with feline infectious peritonitis virus (FIPV) inhibited the protective Th1 cell response by promoting the signaling pathway of IL-10 expression [49]. Natural killer cells are active in the response to numerous infectious diseases and regulate immune response by activating a series of cytokines including IL-12, IL-1β, IL-18, IL-23, and IFN-β, sometimes resulting in hypersensitivity reactions and autoimmune diseases [50]. B cell, CD4^+^ T cells, and CD8^+^ T cells, with migration and secretion features, exert important protective functions during adaptive immune responses in organisms. CD4^+^ helper T cells fight pathogens by activating T-cell-dependent B cells and supporting humoral and cellular immunity. Cytotoxic CD8^+^ T cells kill infected cells using a specific antigen response that corresponds with tissue damage [51]. Due to the antigenic stimulation and activation of antigen-presenting cells and Th cells, activated B cells differentiate into plasma cells and secrete pathogen-specific antibodies to inhibit the effects of pathogens.

Although SARS-CoV-2 is sensitive to cell-surface PRRs, immune evasion is achieved by defending intermediate products of viral replication from immune recognition, resulting in spread of SARS-CoV-2 and restricted immune responses, which are associated with lymphopenia [47, 52]. However, although immune evasion of SARS-CoV-2 temporarily restricts the innate immune response, subsequent overactivation or eruptive initiation of the immune system can occur, leading to multiple organ damage [53]. A hyperactivated immune response contributes to immunopathogenesis, tissue damage, and severe complications. The presence of lymphopenia in 2019-nCoV infection indicates that SARS-CoV-2 affects lymphocytes. Although the CD4^+^ and CD8^+^ T cell levels in peripheral blood are largely decreased, the function of lymphocytes is overactivated. Flow cytometric analysis has indicated high levels of proinflammatory CCR4^+^CCR6^+^ Th17 in CD4^+^ T cells and cytotoxic granules in CD8^+^ T cells, which are associated with systemic inflammatory responses and toxic reactions [54, 55]. In addition, the depressed immune response also indicates the mechanism of immune evasion in SARS-CoV infection [56]. CD3^+^, CD4^+^, and CD8^+^ T lymphocytes were shown to be decreased in the acute phase of SARS-CoV infection, indicating lymphocyte deterioration and a suppressed immune system. Nine hours after the cellular infection of SARS-CoV in vitro, the incomplete viral replication of SARS-CoV led to low production of antiviral cytokines (IFN-α, IFN-β, IFN-γ, and IL-12p40), mild generation of proinflammatory cytokines (TNF-α and IL-6), and significantly elevated inflammatory chemokines (MIP-1a, IP-10, and MCP-1) [57].

Activation of the immune system in response SARS-CoV-2 and subsequent signaling cascades lead to innate and adaptive immunity and proliferation of proinflammatory cytokines, neutralizing antibodies, and recruited lymphocytes, such as neutrophils and macrophages. However, surviving virus excessively stimulates immune cells with positive feedback and causes an inflammatory factor storm. A recent report of 138 patients with SARS-CoV-2 at Zhongnan Hospital of Wuhan University indicated that adverse reactions in severe cases included neutrophilia, coagulation activation, and acute multiple organ injury and that these reactions were associated with higher concentrations of white blood cells and neutrophils, D-dimer, creatine, aspartate aminotransferase, and high-sensitivity troponin I [18]. Another report from Wuhan demonstrated that, compared with healthy people, 44 patients with SARS-CoV-2 had higher immune cytokine counts, including IL-1b, IL-6, IL-12, IFN-γ, IP10, and MCP-1, resulting in systematic toxic organ changes and severe tissue damage [24]. Moreover, patients admitted to the ICU presented with higher levels of GCSF, IP10, MCP-1, MIP1A, and TNF-α [58].

It is believed that the immune response that aims to kill SARS-CoV-2 also disrupts tissue homeostasis and induces immunopathological changes, which is similar to MERS-CoV and SARS-CoV infection [49]. In an analysis of 128 serum samples of SARS patients, T cell responses, especially CD8^+^ T cell responses, and antibody production were found to be major components of the immune response to SARS-CoV infection. The serological manifestation of memory phenotype (CD27^+^/CD45RO^+^) CD4^+^ T cells producing IFN-γ, TNF-α, and IL-2 and CD8^+^ T cells producing IFN-γ, TNF-α, and CD107a was correlated with severe disease. High concentrations of plasma IFN-γ, IL-1β, IL-6, IL-8, IL-12, IP-10, MCP-1, CXCL8, CXCL10, and CCL2 granules are a result of hyperactivated inflammatory signaling cascades and cytokine storm and are associated with the immunopathological changes and severity of SARS-CoV infection [59]. In SARS-CoV infection, neutrophils and chemokines such as IL-8 infiltrate the respiratory tract and generate myeloperoxidase and elastase, which causes deterioration of pulmonary tissue and function and leads to ARDS, respiratory failure, and admission to the ICU. A research investigated 27 serum samples of MERS-CoV from patients from South Korea in 2015 [60]. They found that the CD8^+^ T cell response and proinflammatory factors are associated with severe disease, whereas CD4^+^ T cell response is associated with less severe disease. CD8^+^ T cells act on viral S protein in the early phase of MERS-CoV infection, whereas CD4^+^ T cells interact with E/M/N proteins in the later phase. The invasion of MERS-CoV in host cells triggers the Th1 and Th17 proinflammatory response and stimulates monocytes and lymphocytes, resulting in high levels of IFN-γ, TNF-α, IL-15, and IL-17 and promoting activation of the MAPK, STAT3, and NF-κB signaling pathways. The downstream signaling protein and secreted inflammatory factors fight against the virus, even leading to tissue damage, via the production of IL-6, IL-1β, TGF-β, TNF-α, IL-8, and MCP-1. In addition, an elevated IL-10 level correlates with activated JAK-STAT pathway and indicates an anti-inflammatory effect [61].

Therefore, decreased lymphocytes and the induction of cytokine storm are potent indicators of severe COVID-19 infection. In a study of 228 patients with SARS, patients with severe disease had high levels of IL-6 and reduced concentrations of IL-8 and TGF-β in the acute phase, which correlated with disease severity [62]. The cytokine profiles caused by excessive immune response lead to ARDS and multiple organ failure, contributing to the mortality of patients with COVID-19 [63]. Plasma exchange can clear inflammatory factors, block cytokine storms, and reduce the damage caused by the inflammatory response.

## SARS-CoV-2 and cytokine storms in ICH patients

After mechanical injury by ICH, activated microglia migrate to the position of damage. Although M1 microglia help clear necrotic substances, they also generate inflammatory cytokines and contribute to BBB breakdown and brain edema. Triggered inflammatory cascades, including production of IL-1β, TNF-α, ROS, chemokines, and prostaglandins, damage the BBB [64]. Due to increased BBB permeability, mobilized neutrophils in the perihematomal region generate ROS and release a series of granules, such as collagenase, myeloperoxidase, and elastase. Neutrophils can stimulate nearby microglia, regulate immune response, and exaggerate adverse effects on brain tissue via production of IL-8, IL-6, TNF-α, and IL-1β, resulting in neuronal loss and brain edema. Persistently high neutrophil levels in peripheral blood predict poor prognosis in ICH patients. In addition, neutrophils are important mediators in the recruitment of monocytes [65]. The reactive astrocytes gather around the hematoma and induce MMP-9 [66]. Elevated MMP-9 activity is associated with perihematomal edema, BBB disruption, and neural loss [67]. CD8^+^ cytotoxic T cells and CD4^+^ Th cells increase in the perihematomal region and contribute to neuronal apoptosis and endothelial injury. Due to physical damage and BBB impairment, inflammatory cells infiltrate the hematoma, stimulate the production of cytokines and chemotactic factor with active feedback, and initiate cellular apoptosis via NF-κB inflammatory signaling pathways and downstream molecules [68]. Intercellular adhesion molecule-1, IL-1β, TNF-α, chemokines, MMP-9, inducible nitric oxide synthase, free radicals, COX-2, and PLA_2_ participate in NF-κB activation. Inflammatory cells contain recruited neutrophils and monocytes and resident microglia and astrocytes. Active cytokines can stimulate the complement system to form the membrane attack complex and generate C3a and C5a, resulting in direct tissue injury and augmented immune response. Infiltration of blood substances affects microcirculation, contributing to hypoxia and producing ROS. Hemoglobin and iron are cytotoxic and cause oxidative and proinflammatory changes that further brain injury, probably in conjunction with oxygen free radicals [69]. Oxidative stress, excitotoxicity, and cellular necrosis and apoptosis result in neuronal injury, brain edema, and cell death [70].

However, there is limited information about the innate immune responses in the center nervous system after SARS-CoV-2 brain invasion in ICH patients. In a lab study, the serum samples of SARS-CoV-2 patients were IgM positive in the early stage of infection and subsequently became IgG positive, indicating a humoral response [11]. Because SARS-CoV-2 invades the brain via ACE2 receptor in ICH patients, viral pathogenicity and replication destroy the blood–brain barrier and induce dynamic immune responses. SARS-CoV-2 infection may disturb the activation and inhibition of related signaling cascades, leading to stimulation of the innate immune system, recruitment of lymphocytes, secretion of toxic substances, and cytokine storm with positive feedback circulation.

Neurologic biopsies of patients with SARS-CoV-2 infection demonstrate congestion, brain edema, and partial neuronal degeneration, similar to the effects seen with SARS and MERS infection. ACE2 receptors are distributed throughout the synaptic membrane of the brain, and center nervous system autopsies of SARS patients demonstrated infiltration of monocytes and lymphocytes in blood vessels, hydrocephalus, demyelination of the nerve myelin sheath, and neuronal degeneration, which is associated with aggravation of the pathological changes seen in ICH patients [19]. After SARS-CoV infection in K18-hACE2 mice, viral particles and antigens were found in the neurons of the brain. Upregulation of cytokines and chemokines contributes to BBB impairment, gliocyte hyperplasia, neuronal damage, and brain edema as a result of cellular oxidative damage, necrosis, and apoptosis [71]. Some patients with severe MERS-CoV infection manifested neurological symptoms, including epilepsy, dystaxia, paralysis, and conscious disturbance. Magnetic resonance imaging performed in hospitalized patients with MERS indicated acute alterations in the white matter and the subcortical areas of the frontal, temporal, and parietal lobes [72]. In MERS, excessive production of proinflammatory cytokines and chemokines leads to rapid increases of RIG-I, MDA5, PKR, MYD88, TNF-α, IL-1β, CCL2, CCL5, and CXCL10 in the brain [73]. MHV affects oligodendrocytes and impairs the myelin sheath via immunologic injury. Although MHV-JHM infection in brain tissue initiates an immune response and activates inflammatory signaling cascades to clear the virus, MMP secretion, immunocyte migration, and increased chemokines and cytokines are associated with BBB breakdown and demyelination [49]. Impairment of brain microvascular endothelial cells (BMECs) in vitro by MHV3 infection is a result of downregulation of zona occludens protein 1 (ZO-1), VE-cadherin, and occludin, which leads to elevated BBB permeability [74]. In addition, stimulation and recruitment of macrophages and/or microglia in the white matter contributes to demyelination in MHV-JHM-infected mice. Although immune responses help clear pathogens, excessively inflammatory signaling cascades, influx of cytokines and chemokines, a large volume of recruited immune cells, and toxic substances in the center nervous system indicate a poor prognosis.

Whereas SARS-CoV-2 invasion and replication in brain cause direct damage, indirect deterioration is associated with the immune response. Immune mediator dysfunction and autoimmune reactions prolong the immune response and exacerbate tissue damage. Neutrophils, natural killer cells, macrophages, and lymphocytes proliferate and produce IL-1α, IL-1β, IL-6, IL-12, TNF-α, IFN-γ, and CXCR2. Migrated neutrophils swallow viral particles; generate a series of antibacterial peptides, proteases, and ROS to kill the virus; and introduce tissue damage. ROS, superoxide anion, and NADPH oxidase cause excessive oxidative stress. Elevated neutrophil-to-lymphocyte ratio (NLR) has been shown to be a marker of severe SARS-CoV-2 infection in the early phase.

An overactivated immune system affects both virus and host cells. As SARS-CoV-2 combines with ACE2 receptors, the immunopathological injury in center nerve system is the result of the explosive cytokine storm [75]. ACE2 is highly expressed in arterial and venous endothelial cells and arterial smooth muscle cells in brain. The impairment and contraction of vascular endothelial cells, due to the out of control inflammatory response, lead to increased permeability of the capillary wall and diffusion of substances from vessels into the interstitial space. Brain tissues with ACE2 receptors are attacked the extreme immune response, eventually leading to neurologic deficits and bad outcomes.

## CONCLUSIONS

Patients with COVID-19 who present with neurologic symptoms need early diagnosis, isolation, and treatment. When new neurologic symptoms occur in hospitalized patients, such as ataxia, focal motor deficits, and conscious disturbance, cerebrospinal fluid examination and SARS-CoV-2 nucleic acid and gene sequencing should be performed. ICH patients with SARS-CoV-2 infection are prone to develop neurological complications and have poor outcomes. Because there is no specific treatment for the virus, airborne precautions and isolation of identified and suspected infected patients is crucial.
